# Supplementary material for: Post-traumatic growth experience with kinship hematopoietic stem cells transplantation in patients with aplastic anemia: A qualitative study
Source: PLoS One. 2025 Jul 10;20(7):e0322087. doi: 10.1371/journal.pone.0322087 (PMC12244771; doi:10.1371/journal.pone.0322087)
Supplement: S1 Fig — (DOCX) [file pone.0322087.s001.docx]

**Supporting information**

**S1-1 Fig. Ethics review opinion (Original document）**


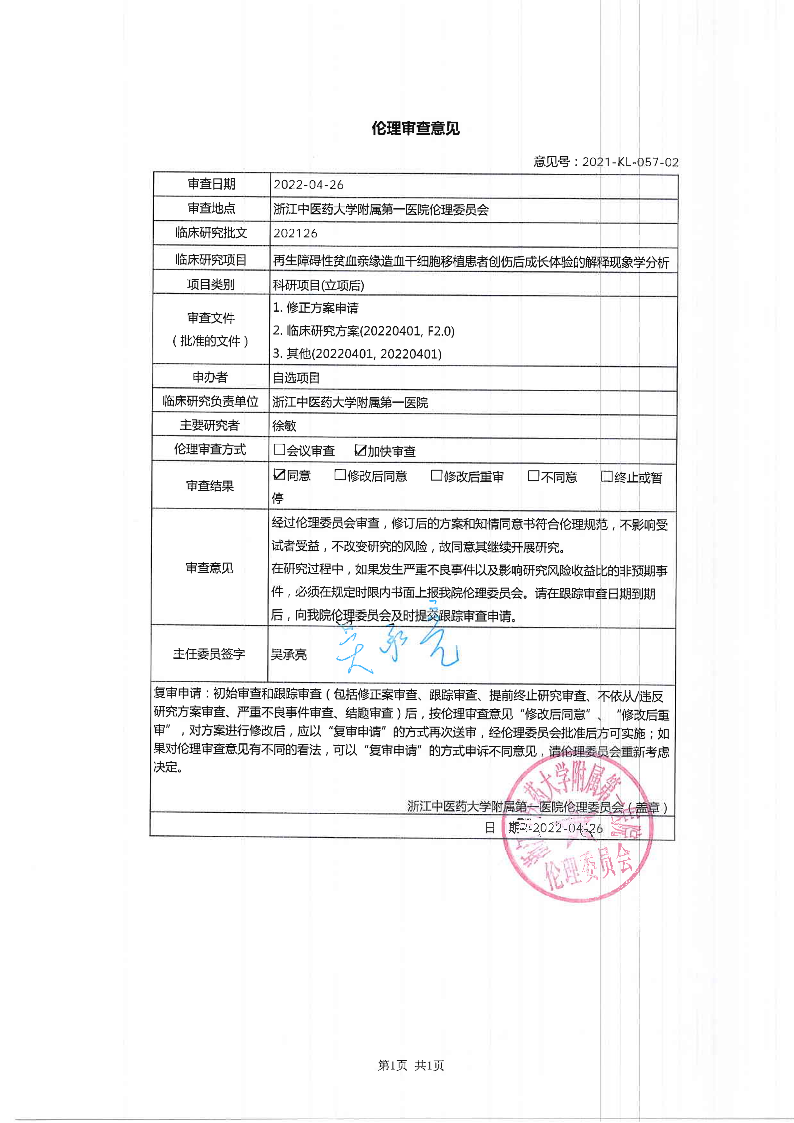


**S1-2 Fig. Ethics review opinion (Translated version）**

**Ethics review opinion**

**Approval Number: 2021-KL-057-02**

| Date of review | 26/04/2022 |
| --- | --- |
| The location of the audit | The Ethics Committee of the First Affiliated Hospital of the Zhejiang Chinese Medical University |
| Clinical research approval | 202126 |
| Clinical research project | Post-traumatic growth experience with kinship hematopoietic stem cells transplantation in patients with aplastic anemia: A qualitative study |
| Project category | Scientific Research Project (after project establishment) |
| Review documents (approval documents) | 1. Application for revision protocol; 2. Clinical Research Protocol (20220401, F2.0); 3. Others (20220401, 20220401) |
| Applicants | Optional project |
| Institution responsible for clinical research | the First Affiliated Hospital of the Zhejiang Chinese Medical University |
| Main researcher | Min Xu |
| Ways of ethical review | Meeting Review √ Speed up the review |
| Results of the audit | √Agree Agree after revision Review after revision  Disagree Terminate or suspend |
| Opinion of the audit | Upon review by the Ethics Committee, the revised protocol and informed consent were in line with ethical norms, did not affect subject benefits, did not change the risks of the study, and were agreed to continue the study.  During the course of the study, serious adverse events and unexpected events that affect the risk-benefit ratio must be reported in writing to the ethics committee within the specified time frame. Please submit the application for follow-up review to the Ethics Committee of our hospital in time after the date of follow-up review expires. |
| Signed by the chairman | Chengliang Wu |
| Request for review: After Initial Review and follow-up review (including amendment review, follow-up review, early termination of study review, non-compliance/non-compliance study programme review, Serious Adverse Event Review, Closure Review) , “Consent after modification” according to the ethical review opinion, “Retrial after revision” after the scheme has been revised, it shall be resubmitted to the Ethics Committee for approval before it can be implemented. If there are different views on the review opinions of the Ethics Committee, the Ethics Committee may appeal for different opinions in the form of a“Retrial application” and request the Ethics Committee to reconsider its decision.  The Ethics Committee of the First Affiliated Hospital of the Zhejiang Chinese Medical University | |
| Date:26/04/2022 | |
