# Supplementary material for: Post-traumatic growth experience with kinship hematopoietic stem cells transplantation in patients with aplastic anemia: A qualitative study
Source: PLoS One. 2025 Jul 10;20(7):e0322087. doi: 10.1371/journal.pone.0322087 (PMC12244771; doi:10.1371/journal.pone.0322087)
Supplement: S2 Table — (DOCX) [file pone.0322087.s002.docx]

**Supporting information**

**S2 Table. Interview outline**

| Interview outline |
| --- |
| 1. Can you tell us about your illness? 2. Under what circumstances did you and your family start considering a kinship transplant? 3. How did you feel when you knew that your family could give you a transplant? 4. What does receiving a transplant from your family mean to you? 5. What do you find most difficult and stressful about receiving a family transplant? How did you cope? 6. What help did you receive during your family transplant? What are the takeaways? 7. What has changed since your family transplant? How did these changes happen? 8. What did you experience/remember the most after receiving a family transplant? 9. How do you feel about your family transplanting you today? 10. What are your plans for the future? How else can you help? |
