# Supplementary material for: Post-traumatic growth experience with kinship hematopoietic stem cells transplantation in patients with aplastic anemia: A qualitative study
Source: PLoS One. 2025 Jul 10;20(7):e0322087. doi: 10.1371/journal.pone.0322087 (PMC12244771; doi:10.1371/journal.pone.0322087)
Supplement: S3 Table — (DOCX) [file pone.0322087.s003.docx]

**S3 File. Informed consent**

**Interpretative Phenomenological Analysis of the Post-traumatic Growth Experience with Kinship Hematopoietic Stem Cells Transplantation in Aplastic Anemia Patients**

Informed Consent Form and Informed Notification Page

Version number：Z2.0

Version date：20220401

Dear Patients,

We will invite you to participate in a study on "An Explanatory Phenomenological Analysis of the Posttraumatic Growth Experience of Associated Hematopoietic Stem Cell Transplant Patients with Aplastic Anemia" to explore the dynamic process of change from trauma to growth in related hematopoietic stem cell transplant patients with aplastic anemia. Before you decide whether or not to participate in this study, please read the following as carefully as possible, it can help you understand the study and why you are conducting it, the procedure and duration of the study, and the benefits, risks, and discomfort that may come to you if you participate in the study. If you wish, you can also discuss it with your relatives, friends, or ask your doctor to explain it and help you make a decision.

**Introduction**

In recent years, the incidence of hematological diseases has increased year by year, with about 75,000 new cases of hematological diseases every year, and the ranking of the cause of death of diseases is gradually increasing, and the threat to human health is becoming increasingly severe. Among them, aplastic anemia, referred to as aplastic aplasia, is a common clinical hematologic disease, which can be divided into severe and non-severe according to the severity of the disease. The incidence of severe aplastic aplastic is dangerous, the prognosis is poor, the mortality rate is high, and the course of non-severe aplastic is long, the cost is large, the burden is heavy, and there is a risk of turning into severe aplasia, which seriously affects the normal work and life of patients. As the only cure for aplastic bone marrow failure, hematopoietic stem cell transplantation (HSCT) has been widely used in hematological diseases and inherited metabolic diseases in recent years.

In traditional allogeneic hemorrhoidentical hematopoietic stem cell transplantation, only 1/4 of patients have a chance of being matched with siblings, while in non-related people, the probability of successful matching of unrelated human leukocyte antigens is less than 1/100,000. With the improvement of transplantation technology, related haploidentical hematopoietic stem cell transplantation has been widely recognized worldwide and has been used in both malignant and non-malignant hematologic diseases. Although related haploidentical transplantation provides more transplant opportunities for patients with aplasia, in addition to the problems caused by HSCT itself, related haploidentical transplantation may cause more serious physical, psychological, and social problems. Due to the combination of the transplant itself and the problems associated with the transplantation, the transplant is undoubtedly a traumatic event for patients with aplastic disease, for which patients with aplastic disease may develop negative emotions such as anxiety, depression, and post-traumatic stress disorder. At the same time, there are also patients with aplastic relatives who have successfully broken through the shackles of body and mind, further explained and accepted themselves in the midst of their illness, reshaped their understanding of the world, further understood the meaning and responsibility of their own existence to their families, bravely embraced a new life, and produced a series of positive psychological changes such as post-traumatic growth (PTG). Therefore, this study intends to use the method of interpretive phenomenological analysis to explore the PTG experience of patients with aplastic kinship transplantation, explore the dynamic change process of patients from trauma to growth, and discover the important trigger points of growth, which can provide reference for similar patients to a certain extent.

The purpose of this study is to solve the occurrence process of PTG experience in patients with aplastic kinship transplantation, to provide an entry point for the development of more complete nursing intervention strategies for patients with aplastic kinship transplantation, and to provide ideas for the psychological rehabilitation of patients after transplantation.

The Ethics Committee has considered that the study is in accordance with the principles of the Declaration of Helsinki and is in line with medical ethics.

**Who should not participate in the study？**

1. Patients whose medical information or related transplant donors are kept confidential;
2. Those who have other major life events in the 1 year before and after HSCT, such as: death of relatives, car accidents, etc.;
3. Those who refuse to record.

**What will need to be done if enrolled in the study？**

1. The source of this study is mainly interviews with study participants, and I will conduct in-depth face-to-face interviews with you;
2. The interview time is about 45-60 minutes, if you feel unwell, you can terminate the interview at any time;

(3) The interview location is of your choice, which can be a quiet and comfortable office, a café or an outdoor location;

(4) The content of the interview mainly involves your psychological feelings and experiences after experiencing hematopoietic stem cell transplantation.

**Possible benefits of participating in the study**

Your participation may not directly benefit you, however, it will have a positive impact on the psychological rehabilitation and care of other aplastic related hematopoietic stem cell transplant patients.

**Possible risks and inconveniences of participating in the study**

The in-depth interview in this study involves your inner thoughts and mental journey after receiving a related hematopoietic stem cell transplant, and the information is relatively sensitive, which may cause you doubts or discomfort. Although no adverse effects have been found in the study so far, if you experience any discomfort during the study, or if there is a new change in your condition, or any unexpected circumstances, please contact the study leader and come to the hospital as soon as possible.

The research group will do its best to prevent and treat possible injuries caused by this study. If there is an adverse event in the clinical study, the government management department and the hospital ethics committee of the research project will determine whether it is relevant to the study. The research group will provide the cost of treatment and corresponding financial compensation for the damage related to the study.

**Fees**

The information gathered in this study is for the purpose of the research project and does not involve any commercial interest. The study will reimburse you for the cost of transportation to participate in the study. In the event of research-related damages, the research team will pay for your medical expenses. If you have other conditions at the same time, the treatments and tests required will not be included in the free offering.

**Is personal information confidential?**

The researcher of this study will strictly abide by the research ethics and keep the interview information confidential, your personal information will be anonymized in the research, if the information you provide is cited in the research report, I will verify the content of the report to you before using it for the final report. Investigators, sponsor representatives, and ethics committees will be allowed access to your profile. Any public report on the results of this study will not reveal your personal identity. We will make every effort to protect the privacy of your personal medical information to the extent permitted by law.

**Where can I get more information?**

You can ask any questions about this study at any time. The study leader will leave you her phone number so that she can answer your questions. If you have any complaints about participating in the study, please contact the Hospital Ethics Committee office. The study leader will keep you informed if there is any important new information during the course of the study that may affect your willingness to continue participating in the study.

**Can voluntarily choose to participate in the study and withdraw from the study halfway**

Whether or not to participate in the study is entirely up to your voluntariness. You may refuse to participate in the study, or withdraw from the study at any time during the course of the study, without affecting your relationship with your physician or with any loss of medical or other benefits to you. You may not participate in the study, or opt out of the study.

**What to do now?**

It's up to you to decide whether or not to participate in this study. You can discuss it with your family or friends before making a decision. Before you make a decision to participate in the study, ask your doctor as many questions as possible until you fully understand the study.

Thank you for reading the above material. If you decide to participate in this study, please let us know and we will arrange everything for you. Please keep this information.

**Informed consent form and consent signature page**

Research Project Title: Interpretative Phenomenological Analysis of the Post-traumatic Growth Experience with Kinship Hematopoietic Stem Cells Transplantation in Aplastic Anemia Patients

Carried out by: The First Affiliated Hospital of Zhejiang University of Traditional Chinese Medicine

Ethics review approval number：2021-KL-057-02

Declaration of Consent

I have read the above presentation of this study and have had the opportunity to discuss and ask questions about this study with my doctor. All the questions I asked were answered satisfactorily.

I am aware of the risks and benefits that may arise from participating in this study. I understand that participation in the study is voluntary, I confirm that I have had sufficient time to consider this, and I understand that:

I. I can ask my doctor for more information at any time.

II. I can withdraw from this study at any time without discrimination or retaliation, and my medical treatment and rights will not be affected.

If I need to take any other medication due to a change in my condition, I will seek the doctor's advice beforehand or tell the doctor truthfully afterwards.

I consent to the access of my research materials by the Ethics Committee or the Sponsor's representatives and the Study Quality Monitor.

I agree to □ or refuse □ other studies other than this study utilize my medical records and pathological examination specimens.

I will be given a signed and dated copy of the informed consent form.

In the end, I decided to agree to participate in this study.

Subject signature： Date:

Subject's contact number：

Contact number of the subject's relatives：

Signature of the legal representative (if any):

I confirm that the details of this study, including its rights and possible benefits and risks, have been explained to the patient and given him a copy of the signed informed consent form. Investigator's signature： Date：

Investigator's contact number：

Contact number of the Office of the Ethics Committee of the First Affiliated Hospital of Zhejiang University of Traditional Chinese Medicine：0571-87072953
