# Supplementary material for: Post-traumatic growth experience with kinship hematopoietic stem cells transplantation in patients with aplastic anemia: A qualitative study
Source: PLoS One. 2025 Jul 10;20(7):e0322087. doi: 10.1371/journal.pone.0322087 (PMC12244771; doi:10.1371/journal.pone.0322087)
Supplement: S4 File — (DOCX) [file pone.0322087.s004.docx]

**S4 File. General Information Questionnaire**

No. Survey time：

This questionnaire is mainly to understand your general situation, your information will be kept confidential, please cooperate with the filling, thank you!

**General Information Questionnaire**

1. Sex：□male □female
2. Age： years old
3. Type of disease：
4. Course of illness： months Duration of transplantation： months
5. Marital status：□married □unmarried □divorced □widowed □privacy
6. Educational level：□elementary school and below □junior high school □high school □college degree or above
7. Primary Carer (Multiple Choices)：□parents □children □spouse □sibling □caregivers □not
8. Religious beliefs：□Not □Yes □Privacy
9. Past medical history：□diabetes □hypertension □Hyperlipidemia □heart disease other □not
10. Current blood transfusion：□Yes □Not
11. Transplant providers：
12. Donor age： years old
13. Degree of HLA conformity：
14. Donor blood type：□Blood type A □Blood type A □Blood type A □Blood type AB
15. Patient's pre-transplant blood type：□Blood type A □Blood type A □Blood type A □Blood type AB
16. Patient's post-transplant blood type：□Blood type A □Blood type A □Blood type A □Blood type AB
17. Transplant Type (Multiple Selections)：□ bone marrow □ peripheral blood □ umbilical cord blood □ mesenchymal stem cells □ others

Thank you very much for participating!
