# Supplementary material for: Post-traumatic growth experience with kinship hematopoietic stem cells transplantation in patients with aplastic anemia: A qualitative study
Source: PLoS One. 2025 Jul 10;20(7):e0322087. doi: 10.1371/journal.pone.0322087 (PMC12244771; doi:10.1371/journal.pone.0322087)
Supplement: S5 File — (DOCX) [file pone.0322087.s005.docx]

**S5 File. Chinese Version Post-Traumatic Growth Rating Index**

**Chinese Version Post-Traumatic Growth Rating Index（C-PTGI）**

The table below has a total of 20 questions about the changes that this transplant may bring to you, please read each sentence carefully, and then correspond to each question, choose the answer that is closest to your situation and mark it "√": 0 is not at all, 1 is very little, 2 is little, 3 is some, 4 is more, and 5 is very much.

| After going through this kinship transplant, I think… | | not at all | very little | little | some | more | very much |
| --- | --- | --- | --- | --- | --- | --- | --- |
| 1 | I changed the order of the important things in my life | 0 | 1 | 2 | 3 | 4 | 5 |
| 2 | I have a greater appreciation of the value of my life (positive recognition) | 0 | 1 | 2 | 3 | 4 | 5 |
| 3 | I developed a new interest | 0 | 1 | 2 | 3 | 4 | 5 |
| 4 | I have a feeling of being more dependent on myself | 0 | 1 | 2 | 3 | 4 | 5 |
| 5 | I have a better understanding of spiritual things | 0 | 1 | 2 | 3 | 4 | 5 |
| 6 | I understand that I can rely on others when I'm struggling | 0 | 1 | 2 | 3 | 4 | 5 |
| 7 | I set a new path in life | 0 | 1 | 2 | 3 | 4 | 5 |
| 8 | I feel closer to others | 0 | 1 | 2 | 3 | 4 | 5 |
| 9 | I prefer to express my emotions | 0 | 1 | 2 | 3 | 4 | 5 |
| 10 | I know I can handle difficulties better | 0 | 1 | 2 | 3 | 4 | 5 |
| 11 | I can do better things with my life | 0 | 1 | 2 | 3 | 4 | 5 |
| 12 | I'm more receptive to the end result of anything | 0 | 1 | 2 | 3 | 4 | 5 |
| 13 | I can cherish every day better | 0 | 1 | 2 | 3 | 4 | 5 |
| 14 | This event has given me new opportunities | 0 | 1 | 2 | 3 | 4 | 5 |
| 15 | I have more empathy for others | 0 | 1 | 2 | 3 | 4 | 5 |
| 16 | I put more effort into relationships | 0 | 1 | 2 | 3 | 4 | 5 |
| 17 | I'm more inclined to change something that needs to be changed | 0 | 1 | 2 | 3 | 4 | 5 |
| 18 | I found myself stronger than I ever imagined | 0 | 1 | 2 | 3 | 4 | 5 |
| 19 | I realized how wonderful people are | 0 | 1 | 2 | 3 | 4 | 5 |
| 20 | I am more accepting of my need for others | 0 | 1 | 2 | 3 | 4 | 5 |
