# Supplementary material for: Post-traumatic growth experience with kinship hematopoietic stem cells transplantation in patients with aplastic anemia: A qualitative study
Source: PLoS One. 2025 Jul 10;20(7):e0322087. doi: 10.1371/journal.pone.0322087 (PMC12244771; doi:10.1371/journal.pone.0322087)
Supplement: S6 Table — (DOCX) [file pone.0322087.s006.docx]

**S6 Table. Preliminary annotations and analysis examples**

| Research participants | Related statements | Preliminary annotations and analysis |
| --- | --- | --- |
| S2 | At first, I was diagnosed with chronic aplastic anemia, but I have been taking traditional Chinese medicine since then. It should have been in the first half of last year, and my blood count is quite good, with platelets exceeding 90000. Later on, it suddenly fell and bled, coinciding with the pandemic. And that drug was cut off during the epidemic. It was inconvenient to come to the hospital during the epidemic. I don't know how many months it was cut off from cyclosporine. Later on, I came over to check the blood count and found out the PNH. The doctor said that you can treat PNH with medication, and medication can suppress it. However, you cannot treat it for a long time, and medication cannot do it for a long time. Later, he said that to control it well, only a transplant can be performed. At the beginning of my blood transfusion, a blood transfusion would stabilize my platelet count at around 60000 yuan. Basically, I would receive a transfusion every two weeks, but later on, I would receive a transfusion for four days because my platelets were also fatigued. After about four days, the blood would quickly fall off. Later on, I had no choice but to confirm the transplant. At that time, the Chinese bone marrow bank doctor also helped me register, but the doctor said that your hope was very slim. One was whether they were willing or not, and the other was that if you want a bone marrow match, the doctor would definitely take 10 points. Because if it is a variant, it requires 10 points, a safety point of 10 points, and for general kinship, it only takes 5 points for you to do it. Is there a blood relationship. Anyway, later on, I couldn't wait any longer. After thinking about it, I decided to do it and just finish it, because it wouldn't be a big deal to waste time. At that time, I didn't think much about myself, mainly children, afraid that they would have an impact. | Before transplantation, the patient's condition cannot be delayed for a long time, and non transplant treatment is an endless wait, so genetic transplantation is the best choice.  Before transplantation, a suitable donor was registered in the Chinese Bone Marrow Bank, but the success rate of matching a completely matched unrelated donor was extremely low, and the hope was slim.  Before transplantation, the Chinese bone marrow bank typing needs to consider the willingness of unrelated donors and whether they will retract midway.  After suffering from PNH, the patient felt extremely reluctant to let go of their child. However, considering accessibility and urgency, they ultimately chose their son's bone marrow.  Fear of causing specific harm to children, detailed description. |
