# Supplementary material for: Post-traumatic growth experience with kinship hematopoietic stem cells transplantation in patients with aplastic anemia: A qualitative study
Source: PLoS One. 2025 Jul 10;20(7):e0322087. doi: 10.1371/journal.pone.0322087 (PMC12244771; doi:10.1371/journal.pone.0322087)
Supplement: S7 Table — (DOCX) [file pone.0322087.s007.docx]

**S7 Table. Examples of basic themes**

| Preliminary annotations and analysis | Primary Theme |
| --- | --- |
| Before the genetic transplant, the patient believed that inserting a tube to draw peripheral blood from such a fragile area around the younger brother's neck was extremely dangerous.（S1）  Before transplantation, the patient's parents were worried that their grandson's bone marrow donation would result in loss of life.（S2）  Before transplantation, patients are concerned about accidents during the transplantation process and the potential impact on the donor's body.（S3）  Before transplantation, patients and mothers were worried that their older sister's body would deteriorate, their resistance would decrease, and they were often prone to illness after donating bone marrow.（S4）  Before the genetic transplant, patients were concerned that donating bone marrow would affect their daughter's future life.（S5）  Before the genetic transplant, patients were afraid that their daughter would suffer from blood related diseases in the future due to bone marrow injury to the foundation.（S5）  Before the genetic transplant, patients were worried that their younger brother's donation of bone marrow would increase the risk of blood disease, and they were afraid of bone marrow dysfunction.（S6）  Before the genetic transplant, patients were worried that their younger brother's lifespan would be shortened, fearing that they would exchange his ten years for her ten years.（S6） | Worried about familial donors |
| Since falling ill, the patient has felt like a burden on her family, and the transplantation of relatives has further increased her sense of guilt.（S1）  Before the genetic transplant, the patient felt guilty because they had implanted something that their child should not have endured at their age.（S2）  After falling ill, the patient's heart is filled with self blame, especially the transplantation of relatives, which has dragged down their family and caused them to be irresponsible.（S2）  My younger sister lost the opportunity to take the college entrance examination due to a genetic transplant, and the patient felt indebted to her.（S3）  Due to the genetic transplant, the patient not only did not bring a better life to their daughter, but also brought disaster to her. The transplant pressure and economic pressure were all on the child's head, and they felt a debt to their daughter.（S5）  Before the genetic transplant, the patient was afraid of burdening her younger brother and wanted to give up, but the brother's determination and courage to save her without hesitation made her feel deeply indebted.（S6） | Genetic transplantation is a debt to family |
