# Supplementary material for: Post-traumatic growth experience with kinship hematopoietic stem cells transplantation in patients with aplastic anemia: A qualitative study
Source: PLoS One. 2025 Jul 10;20(7):e0322087. doi: 10.1371/journal.pone.0322087 (PMC12244771; doi:10.1371/journal.pone.0322087)
Supplement: S8 Table — (DOCX) [file pone.0322087.s008.docx]

**S8 Table. Example of Theme Analysis**

| Theme | Subtheme | Extracting themes | Primary Theme |
| --- | --- | --- | --- |
| Rebirth harvest period | Genetic transplantation brings opportunities for a new life | Reacquiring Life Opportunities | I want to live well after a genetic transplant (S1)  Genetic transplantation is the continuation of life (S2)  Genetic transplantation has given us a chance to revive ourselves, so we should cherish our time and life (S3)  Genetic transplantation is a new life given by my sister (S4)  Genetic transplantation is the process of "regenerating" patients using their daughter's blood (S5)  Genetic transplantation is rebirth of Nirvana (S6) |
|  | Kinship transplantation brings about the reconstruction of relationships | Always harbor a grateful heart | Gratitude to donors for donating bone marrow (S1) after genetic transplantation  After the genetic transplant, I am grateful for all the sacrifices and contributions made by my family, and strive for hard work in return (S3)  Being grateful to the donor after genetic transplantation and striving to repay the "favor" in the future (S4)  Thanks to others for their help after genetic transplantation, continuing to convey great love and kindness (S5)  Gratitude for the family's life-saving kindness after genetic transplantation (S6) |
|  | Genetic transplantation brings personal growth | Enhanced emotional regulation ability | Being better at expressing oneself after genetic transplantation (S1)  Being more accepting of the "bad" outcome of things after genetic transplantation (S2)  After kinship transplantation, actively adjusting from passive acceptance to active acceptance of suffering (S3)  Provide self positive psychological suggestions and quickly adjust emotions after genetic transplantation (S4)  After the transplantation of relatives, the mentality changed from pessimism to optimism (S6) |
