# Supplementary material for: Post-traumatic growth experience with kinship hematopoietic stem cells transplantation in patients with aplastic anemia: A qualitative study
Source: PLoS One. 2025 Jul 10;20(7):e0322087. doi: 10.1371/journal.pone.0322087 (PMC12244771; doi:10.1371/journal.pone.0322087)
Supplement: Attachment — Submitted filename: To the Editor second round.pdf [file pone.0322087.s011.pdf]

From : Luma Hassan Alwan Al Obaidy
University of Baghdad,
College of Science for Women,
Dept. of Biology,
Al –Jadryha
Baghdad,
Iraq.

Dear Editors in PLOS ONE

I hope you are fine.

Thank you to gave me the opportunity to review the manuscript entitled (post-traumatic growth experience with kinship hematopoietic stem cells transplantation in patients with aplastic anemia: A qualitative study).

I would like to inform you that I **Accepted** the amendments and the explanations which are made by the authors, and the final decision is yours.

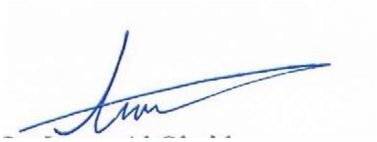

Dr. Luma Hasan Alwan Al Obaidy

1st Aug 2024
