# Supplementary material for: Post-traumatic growth experience with kinship hematopoietic stem cells transplantation in patients with aplastic anemia: A qualitative study
Source: PLoS One. 2025 Jul 10;20(7):e0322087. doi: 10.1371/journal.pone.0322087 (PMC12244771; doi:10.1371/journal.pone.0322087)
Supplement: Attachment — Submitted filename: Response letter.docx [file pone.0322087.s010.docx]

Reviewer 1:

**Q1: The authors state that data underlying the manuscript is fully available, however the supplementary materials do not appear to contain the data, I also haven't been able to find a link to a repository or similar with it stored.**

A: Thanks for the reviewer's advice. There is indeed no supplementary material regarding the data in this study. The available data may be due to a misunderstanding of this option during submission. We apologize for any inconvenience caused.

**Q2: There are sections of the manuscript that need to be edited for clarity, and at times the language used suggests a different interpretation to what I suspect the authors intended. I’ve included some examples here, but feel that the whole manuscript would benefit from revisions.**

A: Thanks for the reviewer's advice. We have made revisions to the details of the article item by item based on expert opinions.

① lines 26-27 last sentence of ‘Background’ needs to be revised for clarity

A: The last sentence of "background" in lines 27-29 has been revised: Understanding the specific psychological changes in a patient's post-traumatic growth experience can help improve the quality of care.

② line 87 The production of PTG in patients helps to alleviate negative psychology

A: The prior sentence in line 87 has been revised: They found that after experiencing traumatic events, in addition to helping alleviate negative psychological experiences, individuals also experience a series of positive psychological changes, such as post-traumatic growth (PTG).

③ lines 104-105 but they have only briefly presented individual areas within the 105 connotation of PTG

A: In response to this issue, we have made modifications to the sentence in lines 107-109: Some current studies have explored the psychological experience of HSCT patients through qualitative research methods, but have not delved into the process of transitioning from trauma to growth from the perspective of AA patients after receiving a relative transplant.

④ line 164 were transcribed into text form by the researcher herself and de-privatized, check for typographical errors e.g., line 382 “sever” should be “severe”

A: We have revised “sever” to “severe” in line 392.

⑤ language when talking about suicide needs revising to be more contemporary e.g., line 490 – unclear if the meaning of the sentence is as the authors intended – S6 appears to have contemplated, rather than committed, suicide

A: We have revised the sentence in line 513～515：S6 felt that her brother seemed to have exchanged his life for her own, leading to suicidal thoughts. The strong psychological fluctuations caused her to pause infusion due to high electrolyte levels that day. The doctor also informed her that her condition was critical.

**Q3: Data Analysis – this section reads more like a how to do IPA than what was done in present study.**

A: Thanks for the reviewer's advice. The process of data analysis was carried out according to the methodology described in the article, and with reference to other similar articles, we made corresponding modifications to the language expression.

**Q4: Table 1 – the information about participants presented here is very detailed, can authors please confirm this can’t be used to identify participants, particularly in conjunction with quotes in Results.**

A: Thanks for the reviewer's advice. We have confirmed that there is no leakage of key patient information and cannot determine the patient based on the content in the table.

**Q5: Results – write up of themes. Can authors clarify what was finding of the present research vs more general comments? it may just be writing style – would expect tense to be past tense, and findings situated in terms of the present study participants, vs more general what tends to happen.**

**Results appears to have discussion-style interpretation woven in e.g., line 238, line 420. There are some valuable observations made here as well as connections to existing research. I suggest removing this information from the Results section and working into the Discussion.**

A: Thanks for the reviewer's advice. ①We have changed some tenses in the Results section to distinguish the general comments from the present research results.

②We have removed discussion-style interpretations from the Results section and working into the Discussion.

**Q6: Discussion-Information here would likely have been better presented in context of the themes, rather than interpreting individual participants circumstances. In my view the Discussion needs re-writing with a greater focus of situating the study findings within the broader PTG post stem cell transplant literature. Bringing in some of the material currently in the Results is my suggestion for a starting point.**

**A:** Thanks for the reviewer's advice. We have rewritten the discussion section to discuss the key aspects of post-traumatic growth for patients with disabilities based on the theme.

**Reviewer 2:**

**Q: I would like you to survey the opinion of care giver a ( nurses , doctors, others.) as a care giver to patients undergo organ transplantation. It will be a complementary study.**

**A:** Thanks for the reviewer's advice. Post traumatic growth is a deep emotional experience within an individual's heart, so when conducting this study, we only designed the research for individual patients. In order to ensure no information bias and explore the post-traumatic growth process of such patients from the perspective of medical staff. We listened to expert opinions, included three medical staff for interviews, and supplemented the content in the main text.
