# Supplementary material for: Post-traumatic growth experience with kinship hematopoietic stem cells transplantation in patients with aplastic anemia: A qualitative study
Source: PLoS One. 2025 Jul 10;20(7):e0322087. doi: 10.1371/journal.pone.0322087 (PMC12244771; doi:10.1371/journal.pone.0322087)
Supplement: Attachment — Submitted filename: 1026Response letter.docx [file pone.0322087.s012.docx]

Dear Reviewers:

I'm sorry that Reviewer 1 didn't see the response letter with detailed information about the changes made. But I am certain that I have uploaded the response letter as requested by the editorial department. This reply letter addresses each item the Reviewer 1 mentioned in your second revision suggestions. Thank you very much for your review!

Q1：Data Analysis – this section reads more like a how to do IPA than what was done in present study.

A1：The specific approach of this study has been indicated in the appendix at the end of the paragraph. For example, in the Preliminary Annotation and Critique section, the end of the paragraph indicates that the specific content about this step in this study is in Table S6.

Q2：Table 1 – the information about participants presented here is very detailed, can authors please confirm this can’t be used to identify participants, particularly in conjunction with quotes in Results.

A2：The patient information provided in Table 1 has been obtained with the patient's consent, and after discussion by the authors, it is unanimously agreed that the patient's privacy will not be disclosed.

Q3：Results – write up of themes. Can authors clarify what was finding of the present research vs more general comments? it may just be writing style – would expect tense to be past tense, and findings situated in terms of the present study participants, vs more general what tends to happen.

A3：The relevant modifications have been completed in the first revised draft. The modified content was to change the tense of the research conclusion of this study to the past tense, and the general conclusion to the present tense.

Q4：Results appears to have discussion-style interpretation woven in e.g., line 238, line 420. There are some valuable observations made here as well as connections to existing research. I suggest removing this information from the Results section and working into the Discussion.
 A4：The proposed modification suggestion has already been modified during the first revision. We removed the original lines 238, lines 420 and so on from the results section and discuss them in the discussion section.

Q5：Discussion

Information here would likely have been better presented in context of the themes, rather than interpreting individual participants circumstances. In my view the Discussion needs re-writing with a greater focus of situating the study findings within the broader PTG post stem cell transplant literature. Bringing in some of the material currently in the Results is my suggestion for a starting point.

A5：The discussion section is divided into paragraphs based on the topic for writing. When discussing arguments, some conclusions about the researcher in the results may be used. Write the content that is not applicable in the Results into the Discussion based on the previous opinion.
