# Supplementary material for: Post-traumatic growth experience with kinship hematopoietic stem cells transplantation in patients with aplastic anemia: A qualitative study
Source: PLoS One. 2025 Jul 10;20(7):e0322087. doi: 10.1371/journal.pone.0322087 (PMC12244771; doi:10.1371/journal.pone.0322087)
Supplement: Attachment — Submitted filename: 3-Response to reviewers.docx [file pone.0322087.s014.docx]

**Response to reviewers**

Thank you to the reviewers for their valuable feedback on this study. After carefully reading the opinions of the experts, our research team members responded to each review comment and made corresponding modifications in the text. The following is the specific content.

1. **Please carefully review the overall manuscript to update the language and provide definitions as needed for specificity and clarity. Examples of unclear language and/or undefined terms include but are not limited to: "transplantation in the warehouse", "entered the transplant bin", "entering the barn," "period of rebirth harvesting."**

Thank you for your valuable suggestion. Based on this opinion, we have re examined the language that may require further clarification of concepts throughout the entire text. For the first three phrases mentioned in the revision suggestions, we believe that the main reason for the unclear definition may be the inconsistent use of words. Therefore, we will unify 'bin' and 'barn' in the text as 'warehouse'. However, the term 'period of rebirth harvesting' is a summary based on qualitative interviews, and the explanation for this period has been elaborated in detail in the following article.

2. **Please provide a rationale for the inclusion of interviews with healthcare workers, as well as more information about the recruitment of healthcare workers in the Methods Section.**

Thank you for your valuable suggestion. Interviewing this group of people was based on the opinions provided by the previous round of reviewers. After discussion, we believe that healthcare workers, as a group of people who closely connect with patients during transplantation treatment, may have a direct understanding of the patient's emotional journey during this special period. In order to gain a more comprehensive understanding of patients' psychological changes, we have decided to conduct additional interviews with healthcare workers as a group. We have explained this opinion in the article and supplemented the inclusion and exclusion criteria for healthcare workers.

3. **Ther inclusion criteria includes "experienced positive changes brought by the event of a kinship transplantation" Please provide a defintion for a positive experience and specify how this was determined.**

Thank you for your valuable suggestion. In this study, we included a population with positive experiences, which mainly refer to positive experiences of post-traumatic growth. This definition has been presented in the introduction section.

4. **The statement on lines 611 - 614 may be unsupported as written should be rephrased. If the statement represents a summary of patient responses please ensure it is supported by direct quotations.**

Thank you for your valuable suggestion. This section is based on the patient's true oral account, rather than references.

5. **Please include a limitations section.**

Thank you for your valuable suggestion. This section has been added to the main text.
