# Supplementary material for: Post-traumatic growth experience with kinship hematopoietic stem cells transplantation in patients with aplastic anemia: A qualitative study
Source: PLoS One. 2025 Jul 10;20(7):e0322087. doi: 10.1371/journal.pone.0322087 (PMC12244771; doi:10.1371/journal.pone.0322087)
Supplement: Attachment — Submitted filename: Reviewer Comments PONE-D-23-37733.pdf [file pone.0322087.s009.pdf]

Thank you for the opportunity to review this manuscript. The focus of the study is of value, and presents an interesting addition to the growing literature about post traumatic growth following kinship hematopoietic stem cells transplantation in patients with aplastic anemia.

The authors state that data underlying the manuscript is fully available, however the supplementary materials do not appear to contain the data, I also haven't been able to find a link to a repository or similar with it stored.

There are sections of the manuscript that need to be edited for clarity, and at times the language used suggests a different interpretation to what I suspect the authors intended. I've included some examples here, but feel that the whole manuscript would benefit from revisions.

- lines 26-27 last sentence of 'Background' needs to be revised for clarity
- line 87 The production of PTG in patients helps to alleviate negative psychology
- lines 104-105 but they have only briefly presented individual areas within the 105 connotation of PTG
- line 164 were transcribed into text form by the researcher herself and de-privatized,
- check for typographical errors e.g., line 382 "sever" should be "severe"
- language when talking about suicide needs revising to be more contemporary e.g., line 490 – unclear if the meaning of the sentence is as the authors intended – S6 appears to have contemplated, rather than committed, suicide

Data Analysis – this section reads more like a how to do IPA than what was done in present study

Table 1 – the information about participants presented here is very detailed, can authors please confirm this can't be used to identify participants, particularly in conjunction with quotes in Results

Results
Results – write up of themes. Can authors clarify what was finding of the present research vs more general comments? it may just be writing style – would expect tense to be past tense, and findings situated in terms of the present study participants, vs more general what tends to happen.

Results appears to have discussion-style interpretation woven in e.g., line 238, line 420. There are some valuable observations made here as well as connections to existing research. I suggest removing this information from the Results section and working into the Discussion.

Discussion
Information here would likely have been better presented in context of the themes, rather than interpreting individual participants circumstances. In my view the Discussion needs re-writing with a greater focus of situating the study findings within the broader PTG post stem cell transplant literature. Bringing in some of the material currently in the Results is my suggestion for a starting point.
